# Specific manifestations of knee osteoarthritis predict depression and anxiety years in the future: Vancouver Longitudinal Study of Early Knee Osteoarthritis

**DOI:** 10.1186/s12891-020-03496-8

**Published:** 2020-07-16

**Authors:** Eric C. Sayre, John M. Esdaile, Jacek A. Kopec, Joel Singer, Hubert Wong, Anona Thorne, Ali Guermazi, Savvas Nicolaou, Jolanda Cibere

**Affiliations:** 1 Arthritis Research Canada, 5591 No. 3 Road, Richmond, BC V6X 2C7 Canada; 2 Medicine, University of British Columbia, Vancouver, BC Canada; 3 Medicine, University of Calgary, Calgary, AB Canada; 4 School of Population & Public Health, University of British Columbia, Vancouver, BC Canada; 5 Radiology, Boston University School of Medicine, Boston, MA USA; 6 Radiology, University of British Columbia, Vancouver, BC Canada

**Keywords:** Osteoarthritis (OA), Depression, Anxiety, Magnetic resonance imaging (MRI)

## Abstract

**Background:**

To evaluate whether knee osteoarthritis (OA) manifestations predict depression and anxiety using cross-sectional and longitudinal prediction models.

**Methods:**

A population-based cohort (*n* = 122) with knee pain, aged 40–79, was evaluated at baseline, 3 and 7 years. Baseline predictors were: age decade; sex; BMI ≥ 25; physical exam knee effusion; crepitus; malalignment; quadriceps atrophy; flexion; flexion contracture; Kellgren-Lawrence (KL) x-ray grade (0/1/2/3+); WOMAC pain ≥25; WOMAC stiffness ≥25; self-reported knee swelling; and knee OA diagnosis (no/probable/definite). Depression and anxiety, cutoffs 5+ and 7+ respectively, were measured via the Hospital Anxiety and Depression Scale. We fit logistic models at each cycle using multivariable models selected via lowest Akaike’s information criterion.

**Results:**

Baseline depression model: sex (female OR = 0.27; 0.10, 0.76) and KL grade (KL 1 OR = 4.21; 1.31, 13.48). Three-year depression model: KL grade (KL 1 OR = 18.92; 1.73, 206.25). Seven-year depression model: WOMAC stiffness ≥25 (OR = 3.49; 1.02, 11.94) and flexion contracture ≥1 degree (OR = 0.23; 0.07, 0.81). Baseline anxiety model: knee swelling (OR = 4.11; 1.51, 11.13) and age (50–59 vs. 40–49 OR = 0.31 [0.11, 0.85]; 60–69 OR = 0.07 [0.01, 0.42]). Three-year anxiety model: WOMAC stiffness ≥25 (OR = 5.80; 1.23, 27.29) and KL grade (KL 1 OR = 6.25; 1.04, 37.65). Seven-year anxiety model: sex (female OR = 2.71; 0.87, 8.46).

**Conclusion:**

Specific knee OA-related manifestations predict depression and anxiety cross-sectionally, 3 years in the future, and for depression, 7 years in the future. This information may prove useful to clinicians in helping to identify patients most at risk of present or future depression and anxiety, thus facilitating preemptive discussions that may help counter that risk.

## Background

Osteoarthritis (OA) is a highly prevalent, disabling and costly condition. In the province of British Columbia, Canada, 11% of the adult (18+) population were reported to have OA in a study evaluating administrative data [1]. In the U.S., clinical OA (defined on the basis of symptoms and physical examination findings) was seen in 27 million adults in 2008 [2]. Symptomatic radiographic knee OA affects 9.5% of elderly adults aged 63 years and older [3]. OA is more prevalent with older age and in obese people and thus, OA constitutes an increasing public health burden. In Canada, the total economic burden for OA, including direct and indirect costs, was estimated at $27.5 billion in 2010, and the cumulative economic burden between 2010 and 2040 is estimated to be $1.45 trillion [4].

Depression and anxiety are common in OA [5], and it would be valuable to identify those most at risk amongst a clinician’s patients, as such foreknowledge would facilitate preemptive discussions that could counter that risk. Unfortunately, within the body of research on depression/anxiety in OA populations (both knee OA and in other joints), relatively few have investigated the specific predictors of depression/anxiety in OA [6–19], and the majority have been cross-sectional. Only four studies investigated possible predictive associations between present-day OA and *future* depression or anxiety, with predictors including number of painful joints, gait speed, pain, disability and fatigue [12, 15, 16, 19]. In the present study, we aim to develop models predicting both present-day and future depression or anxiety from a much larger set of knee OA-related factors easily accessible to clinicians. This study is done on a population-based cohort with knee pain (mild to severe), and includes subjects with radiographic OA, as well as those with pre-radiographic OA, the latter of which tends to be undiagnosed OA in general practice. Since many individual aspects of the OA disease process may be present in a subject without the OA diagnosis itself—for example stiffness, knee swelling or low-grade osteophytes—this therefore represents an expansion of the target population potentially impacted by our findings, beyond simply those with an OA diagnosis.

## Methods

### Study population

The cohort was recruited between 2002 and 2005 and has been described previously in Cibere et al. [20] In brief, 255 subjects aged 40–79 with knee pain were recruited as a population-based random sample in Vancouver, Canada. Stratified sampling was used in order to achieve equal representation within age decades and gender. The pain criteria for inclusion specifically were, “pain, aching, or discomfort in or around the knee on most days of the month at any time in the past” [20], and “any pain, aching, or discomfort in or around the knee in the past 12 months” [20]. While all subjects had knee pain, not all had osteoarthritis. Based on radiograph and magnetic resonance imaging (MRI) cartilage scores, subjects were classified into three subgroups: “no OA” on both MRI and x-ray (both normal) (13%), “pre-radiographic OA” on an abnormal MRI (but normal x-ray) (49%), and “radiographic OA” on an abnormal x-ray (38%). Subjects were excluded both at baseline and/or at follow-up if any of the following applied, as described in Cibere et al. [20]: inflammatory arthritis (or fibromyalgia), knee arthroplasty, knee injury or surgery within the past 6 months, knee pain referred from hips or back, or unable to undergo MRI. Of 255 subjects seen at baseline, 122 subjects successfully completed the second follow-up. Details about reasons for dropout have been described previously in Sayre et al. [21].

### Outcomes and predictor variables

Depression and anxiety were measured via the Hospital Anxiety and Depression Scale (HADS), and the binary outcome variables were based on HADS scales measuring 5+ for depression and 7+ for anxiety [22–24].

Predictor variables related to OA were selected a priori by the study team for clinically relevant items from the literature, or those thought to be clinically relevant, from a much larger set of variables that had been collected on these subjects. We focused on items that are easily accessible to clinical practitioners (such as those measured during a clinical exam, collected via self-reported questionnaire, or scored on a plain radiograph), and/or easily noticeable by (and potentially worrisome to) patients. We identified three major sources of such potential predictors evaluated at baseline.

The first source of potential predictors was derived from a baseline physical examination of the knee. These variables have all previously demonstrated good reliability according to an inter-rater intraclass correlation coefficient (ICC) of at least 0.8 [25]. Data included: alignment by visual inspection (normal/varus/valgus; ICC = 0.94); effusion (present/absent; ICC = 0.97); flexion (degrees; ICC = 0.85); flexion contracture (degrees; ICC = 0.95); crepitus (none/fine/coarse; ICC = 0.96); quadriceps atrophy (none/mild/severe; ICC = 0.97). Alignment was entered into the models as any malalignment vs. normal alignment. Flexion was dichotomized as > = 135 vs. < 135 degrees in the models for ease of interpretation and application by clinicians, who don’t use a goniometer to measure flexion; > = 135 was chosen as the cut point due to its representing “normal” flexion. Similarly, flexion contracture was dichotomized as > = 1 vs. < 1 degree in the models for ease of interpretation and application by clinicians; > = 1 was chosen as the cut point due to its representing the presence of a flexion contracture. Quadriceps atrophy was entered into the models as any atrophy vs. none (i.e., normal).

The second source was derived from a self-reported questionnaire at baseline. Data included: knee swelling in the past 12 months (yes/no); painful joint count using a homunculus; and a previous physician’s diagnosis of study knee OA (no/probable/definite). For knee-related questions, information on the study knee was used in the analysis. In subjects with bilateral pain, the worse (more painful) knee was used as the study knee. Subjects also completed the Western Ontario and McMaster Universities (WOMAC) Osteoarthritis Index VA3.1 [26]. WOMAC scales for pain and stiffness were normalized to a 0–100 range (higher worse), and dichotomized for ease of interpretation and application by clinicians as 25+ vs. < 25 in the analysis; 25 was chosen as the cutpoint for pain based on the univariate distribution (75th percentile), and was also used for stiffness for consistency as it was numerically close to that 75th percentile.

The third source was derived from an x-ray at baseline, obtained using a weight-bearing fixed-flexion posteroanterior view with the SynaFlexer (BioClinica Inc., Newark, CA) positioning frame, and a skyline view in the supine position [27]. Radiographs were read blinded to clinical information, and read and scored by two independent readers for Kellgren-Lawrence (KL) 0–4 grading [28]. Previous studies using these data have demonstrated good interrater reliability (ICC = 0.79) [29]. Differences in readings were adjudicated by consensus readings with both readers. Baseline KL grade was collapsed into 0, 1, 2 or 3+ in the analysis. KL grades 3 and 4 were collapsed due to distribution—only 6% had KL grade 4 at baseline.

Finally, age decade, sex and body mass index (BMI) ≥25 (representing overweight) were included as potential predictors.

### Statistical analysis

Baseline data were summarized using frequencies or means (+ standard deviation), stratified separately by baseline depression and anxiety. Logistic regression was used to model binary depression and anxiety at baseline, as well as at first and second follow-up. In the follow-up models for depression, those with depression at baseline were excluded. Similarly, in the follow-up models for anxiety, those with anxiety at baseline were excluded. Multivariable models were selected via lowest Akaike’s information criterion (AIC), with the additional restriction of *p*-values < alpha = 0.15. The *p*-value < 0.15 restriction was not a pre-screening, but rather imposed during selection, and was meant to prevent possible instability in the model if variables less significant than that were included. Odds ratios (ORs) along with 95% confidence intervals (CIs) were computed from each selected model. Model fit was assessed via the Hosmer and Lemeshow goodness of fit test, which is based on the agreement between observed and predicted probabilities [30]. In addition to selecting models on the basis of predictive utility via lowest AIC, the predictive utility of each selected multivariable model was also assessed via area under the receiver operating characteristic (ROC) curve (AUC), which were reported along with their 95% confidence intervals.

For consistency, the primary analysis utilized the common sample that was successfully followed to 7.5 years in all three analyses (baseline, 3-year and 7.5-year models). However, this could raise the question about a possible survival bias. To assess whether this may have played a role in any observed associations, we performed additional sensitivity analyses in which the selected multivariable models at first follow-up and baseline were re-fit using all available data for their respective time points, rather than only the smaller sample followed to 7.5 years. In addition to this, we compared baseline covariates as well as baseline and 3-year follow-up depression and anxiety in the portion of the baseline sample that was followed up to 7.5 years vs. the portion that was not, via chi-square tests for categorical variables, or t-tests for continuous variables.

To obtain population-based estimates, all analyses were performed using age decade-gender stratum sampling weights [20]. This was required as the original sample was collected in a manner to ensure adequate sample sizes across all in-scope age decade-gender groups (namely 40–79 male/female), for example older age decades were over-sampled and younger decades under-sampled. Descriptive statistics are also reported using the sampling weight, for population representativeness and to reflect the data used in the models.

All analyses were performed using SAS v9.4 (SAS Institute, Cary, North Carolina).

## Results

This cohort study included 122 subjects with data at baseline and follow-up. The median follow-up time was 3.2 years at first follow-up (range 2.5 to 5.1), and 7.5 years at second follow-up (range 6.0 to 9.5). Sample-weighted baseline characteristics, stratified separately by baseline depression and anxiety, are shown in Table 1. Non-depressed (weighted *N* = 97.0), compared to depressed subjects (weighted *N* = 25.0), differed significantly in proportion female (61.4% vs. 33.9%) and KL grade. Non-depressed subjects had substantially fewer KL grade 1 knees (14.4% vs. 40.2%), more KL grade 2 (25.7% vs. 10.0%), and similar proportions with KL grades 0 (42.0% vs. 35.7%) and 3+ (17.9% v. 14.1%). The non-depressed vs. depressed cohorts were similar on mean age (55.5 years vs. 55.8 years) and age decade, and all other assessed variables (Table 1). Non-anxious (weighted *N* = 90.0), compared to anxious (weighted *N* = 32.0), differed significantly on flexion contracture (mean 1.8 degrees vs. 0.6 degrees), self-reported knee swelling (51.4% vs. 48.5%), and age decade (the anxious cohort tended to be younger). However, the non-anxious vs. anxious cohorts did not differ significantly on mean age (56.5 years vs. 52.9 years) and all other assessed variables (Table 1).

**Table 1:** Sample-weighted baseline characteristics

	No baseline depression (Weighted *N* = 97.0)	Baseline depression (Weighted *N* = 25.0)	No baseline anxiety (Weighted *N* = 90.0)	Baseline anxiety (Weighted *N* = 32.0)
Age, mean (SD)	55.5 (9.0)	55.8 (9.9)	56.5 (8.8)	52.9 (9.0)
Age decade, n (%)				
40–49	21.6 (22.3)	9.0 (36.1)	16.4 (18.2)	14.2 (44.5)
50–59	47.8 (49.3)	6.7 (26.7)	41.7 (46.3)	12.8 (39.9)
60–69	17.8 (18.4)	5.1 (20.2)	21.4 (23.7)	1.5 (4.8)
70–79	9.8 (10.1)	4.2 (17.0)	10.6 (11.8)	3.5 (10.8)
Female, n (%)	59.5 (61.4)	8.5 (33.9)	48.8 (53.8)	19.5 (61.1)
Body mass index, mean (SD)	25.9 (4.2)	26.5 (3.3)	26.2 (4.0)	25.7 (4.1)
BMI ≥ 25, n (%)	49.9 (51.4)	17.4 (69.6)	51.5 (57.2)	15.8 (49.4)
Effusion, n (%)	17.9 (18.5)	6.0 (24.0)	16.1 (17.9)	7.8 (24.6)
Crepitus, n (%)				
None	29.2 (30.2)	8.5 (34.0)	27.0 (29.9)	10.8 (33.8)
Fine	49.6 (51.1)	11.9 (47.4)	45.2 (50.2)	16.3 (50.9)
Coarse	18.2 (18.7)	4.6 (18.5)	17.9 (19.9)	4.9 (15.3)
Malalignment, n (%)	11.5 (11.9)	4.6 (18.3)	10.7 (11.9)	5.4 (16.9)
Quadriceps atrophy, n (%)	30.5 (31.4)	7.5 (30.0)	25.2 (27.9)	12.8 (40.1)
Flexion (degrees), mean (SD)	134.3 (8.6)	133.2 (6.9)	134.4 (8.0)	133.2 (8.8)
Flexion contracture (deg.), mean (SD)	1.5 (3.9)	1.7 (2.8)	1.8 (3.9)	0.6 (2.7)
Kellgren-Lawrence grade, n (%)				
0	40.8 (42.0)	8.9 (35.7)	36.1 (40.1)	13.6 (42.6)
1	14.0 (14.4)	10.0 (40.2)	17.1 (19.0)	6.9 (21.6)
2	24.9 (25.7)	2.5 (10.0)	19.9 (22.1)	7.5 (23.5)
3+	17.3 (17.9)	3.5 (14.1)	16.9 (18.8)	3.9 (12.3)
WOMAC pain, mean (SD)	18.0 (16.4)	23.4 (19.9)	18.1 (16.5)	21.9 (19.5)
WOMAC pain (25+)	23.8 (24.6)	8.4 (33.7)	22.6 (25.1)	9.7 (30.4)
WOMAC stiffness, mean (SD)	2.4 (2.4)	2.1 (1.9)	2.3 (2.4)	2.3 (2.0)
WOMAC stiffness (25+)	38.1 (39.3)	11.1 (44.5)	35.7 (39.6)	13.6 (42.4)
Self-reported knee swelling in last 12 months, n (%)	54.8 (56.5)	16.5 (66.0)	46.3 (51.4)	25.1 (48.5)
Painful joint count, mean (SD)	7.8 (6.8)	9.4 (12.3)	7.5 (7.0)	9.8 (11.4)
Previous physician diagnosis knee OA				
No	57.1 (58.9)	16.3 (65.4)	56.6 (62.9)	16.8 (52.7)
Probable	30.4 (31.3)	6.1 (24.4)	24.9 (27.7)	11.6 (36.2)
Definite	9.5 (9.8)	2.6 (10.3)	8.5 (9.5)	3.6 (11.2)

WOMAC = Western Ontario and McMaster Universities (WOMAC) Osteoarthritis Index VA3.1

Table 2 lists the adjusted odds ratios from the selected prediction models for depression. The selected model for baseline depression (AUC 0.722; 95% CI = 0.624, 0.819) included sex (female OR = 0.27; 0.10, 0.76) and KL grade (KL 1 [pre-radiographic OA] OR = 4.21; 1.31, 13.48). The 3.2-year depression model (AUC 0.742; 95% CI = 0.622, 0.862) included KL grade (KL 1 OR = 18.92; 1.73, 206.25). The 7.5-year depression model (AUC 0.606; 95% CI = 0.487, 0.725) included WOMAC stiffness ≥25 (OR = 3.49; 1.02, 11.94) and flexion contracture ≥1 degree (OR = 0.23; 0.07, 0.81).

**Table 2:** Odds ratios in selected multivariable models predicting depression

Parameter	Baseline OR (95% CI)	First follow-up OR (95% CI)	Second follow-up OR (95% CI)
Female	0.27 (0.10, 0.76)	–	–
Kellgren-Lawrence grade			
1 vs. 0	4.21 (1.31, 13.48)	18.92 (1.73, 206.25)	–
2 vs. 0	0.45 (0.09, 2.19)	1.49 (0.07, 33.60)	–
3+ vs. 0	0.64 (0.15, 2.72)	2.50 (0.11, 54.96)	–
Malalignment	4.38 (1.08, 17.77)	–	–
WOMAC stiffness (25+)	–	–	3.49 (1.02, 11.94)
Flexion contracture (degrees) ≥1	–	–	0.23 (0.07, 0.81)

Western Ontario and McMaster Universities (WOMAC) Osteoarthritis Index pain 0–100 scale

- = Not in selected model

Table 3 lists the adjusted odds ratios from the selected prediction models for anxiety. The selected model for baseline anxiety (AUC 0.749; 95% CI = 0.653, 0.846) included age decade and knee swelling (OR = 4.11; 1.51, 11.13). 50–59 and 60–69 age groups were protective vs. 40–49: 50–59 OR = 0.31 (0.11, 0.85); 60–69 OR = 0.07 (0.01, 0.42). The 3.2-year anxiety model (AUC 0.739; 95% CI = 0.622, 0.856) included WOMAC stiffness ≥25 (OR = 5.80; 1.23, 27.29) and KL grade (KL 1 OR = 6.25; 1.04, 37.65). The 7.5-year anxiety model (AUC 0.587; 95% CI = 0.490, 0.685) included sex (female OR = 2.71; 0.87, 8.46).

**Table 3:** Odds ratios in selected multivariable models predicting anxiety

Parameter	Baseline OR (95% CI)	First follow-up OR (95% CI)	Second follow-up OR (95% CI)
Age decade			
50–59 vs. 40–49	0.31 (0.11, 0.85)	–	–
60–69 vs. 40–49	0.07 (0.01, 0.42)	–	–
70–79 vs. 40–49	0.32 (0.07, 1.41)	–	–
Self-reported knee swelling last 12 months	4.11 (1.51, 11.13)	–	–
WOMAC stiffness (25+)	–	5.80 (1.23, 27.29)	–
Kellgren-Lawrence grade			
1 vs. 0	–	6.25 (1.04, 37.65)	–
2 vs. 0	–	0.70 (0.08, 6.16)	–
3+ vs. 0	–	1.95 (0.29, 13.18)	–
Female	–	–	2.71 (0.87, 8.46)

Western Ontario and McMaster Universities (WOMAC) Osteoarthritis Index pain 0–100 scale

- = Not in selected model

In sensitivity analyses in which the selected multivariable models at baseline were re-fit using all available data for that time point (*N* = 255), the directions of the significant associations in the baseline and first follow-up models remained consistent across the depression and anxiety models. Finally, in the comparison of baseline covariates as well as baseline and 3-year follow-up depression and anxiety in the portion of the baseline sample that was successfully followed up to 7.5 years vs. the portion that was not, those who were followed had significantly more self-reported swelling (58.5% vs. 43.8%, *p* = 0.019). There were no other significant differences.

All selected multivariable models passed the Hosmer and Lemeshow goodness of fit test.

## Discussion

We developed prediction models for depression and anxiety (current, 3-year and 7.5 years out) from a variety of easily accessible potential predictors including those sourced from physical examination, self-reported questionnaire, and radiographic evaluation, in a longitudinal population-based cohort with knee pain.

Cross-sectionally, male sex and malalignment were predictive of depression, as was KL grade 1 when compared to grade 0. Grades 2 and 3+ were not significant. One seemingly plausible explanation for this unusual pattern is adaptation to the diagnosis by the time advanced disease sets in. At first follow-up, the same pattern of association is seen vs. KL grade, with no other predictors in the model. At second follow-up, WOMAC stiffness ≥25 was predictive of depression, while presence of a flexion contracture was protective. Cross-sectionally, predictors of anxiety included age decade (50–59 and 60–69 age decades were protective compared to 40–49), and self-reported knee swelling (harmful). That self-reported knee swelling would induce anxiety is not surprising. At first follow-up, baseline predictors of anxiety included WOMAC stiffness (positive association), and KL x-ray grade, with essentially the same pattern as that observed in the depression models. At second follow-up, only female sex was predictive of anxiety, though it was not significant.

The previous literature on this topic is somewhat scant. Of the studies we identified that investigated OA-related predictors of depression or anxiety [6–19], the majority were cross-sectional, and/or explored only the gross association between OA disease as a whole and depression or anxiety. Four studies investigated possible OA-related predictors of future depression or anxiety. Peruccio et al. [12] reported significant positive associations between the number of painful joints preoperatively, and HADS depression and anxiety scales 1 year post-operatively, in a population undergoing knee replacement surgery for OA. Gandhi et al. [13] also reported a positive association between painful joint count and (present) depression and anxiety in a cohort about to undergo knee or hip replacement for OA. In our study, painful joint count was not predictive. It is possible this is due to the cohort entry criteria, which included “pain, aching, or discomfort in or around the knee on most days of the month at any time in the past” (i.e., all subjects had some history of persistent knee pain). In another cohort of preoperative knee and hip osteoarthritis, Wood et al. [11] modelled (present) depression and anxiety versus pain severity, Oxford Score, and KL grade, adjusting for other covariates. The only significant predictor of anxiety they identified was Oxford Score, while for depression both pain and Oxford were predictive. Partially in line with this, we identified WOMAC stiffness as a significant predictor of (future) anxiety and depression.

On the other hand, in a non-preoperative study, Hawker et al. [19] found that disability and fatigue predicted future depression in OA, while pain was not predictive. Similarly, White et al. [16] reported slow gait speed as predictive of worsening depressive symptoms in knee OA. Pain was also not predictive in our multivariable models. Touching on another of our findings, Brandt et al. [17] found a significant relationship between knee pain without OA diagnosis and depression, yet they did not observe this within OA diagnosed subjects. This is in line with our findings with KL grade 1 being predictive, while grades 2 and 3+ are not (again, possibly due to adaptation). Finally, Shimura et al. [18] reported an association between serum interleukin 6 (an inflammatory cytokine) and depressive state, which may be analogous to our finding that self-reported knee swelling cross-sectionally predicts anxiety.

The strengths and limitations of our study deserve comment. While population-based is a strength, it should also be noted that the target population is not the overall population, but those with baseline knee pain, aged 40–79 at baseline, who were successfully followed up over an average of 7.5 years. As such, we cannot be sure that the results of this study are applicable to a more general population. However, considering our objective was to develop prediction models for anxiety and depression from manifestations of OA (an inherently painful disease), this restriction should not be too impactful, and further, our inclusion of mild but persistent knee pain without diagnosed OA actually presents an *expanded* target population compared to some of the comparable literature discussed above. Another strength of this study is a priori selected potential predictors based on literature as well as subject matter expertise. Another limitation of this study is loss to follow-up: only 122 of the original 255 baseline subjects were successfully followed through to the third cycle. To explore the effect this might have had, we analyzed potential differences between those followed to 7.5 years vs. not followed to 7.5 years on all the covariates included in this analysis as well as baseline and 3-year follow-up depression and anxiety, and found no significant differences amongst depression and anxiety or those variables included in the final selected models, except self-reported swelling. Furthermore, in sensitivity analyses, models fit on the larger samples available at each time point were generally consistent with the primary results. It should be acknowledged that data not missing at random (and the bias that could result) may have impacted the models. For example, if subjects with severe OA (advanced KL grade) who were depressed at follow-up were less likely to provide follow-up data than those who were not depressed, then this could produce a conservative bias towards the null. It is also conceivable that other biases could be liberal in direction, for example, if subjects with severe OA who were depressed at follow-up were more likely to provide follow-up data than their non-depressed counterparts (less plausible). Finally, the point estimates reported in this study should be interpreted with some caution, considering the width of many of the confidence intervals. Wide confidence intervals may have resulted from small cell sizes, for example, KL grade 3+ with depression at first follow-up contained only three subjects, producing a particularly wide confidence interval.

## Conclusions

We have developed cross-sectional and longitudinal (at 3 and 7 years) prediction models for depression and anxiety from specific manifestations of OA, drawing from a broad array of potential predictors from a variety of sources (i.e., physical exam, self-reported questionnaire, and radiographic evaluation), in a population with knee pain with or without an OA diagnosis. These models may prove useful to clinicians in helping to identify patients most at risk of either present or future depression and anxiety, thus facilitating preemptive discussions that may help counter that risk.

## Data Availability

The datasets used and analyzed during the current study are available from the corresponding author on reasonable request.

## Abbreviations

OA: Osteoarthritis
MRI: Magnetic resonance imaging
HADS: Hospital Anxiety and Depression Scale
ICC: Intraclass correlation coefficient
WOMAC: Western Ontario and McMaster Universities
KL: Kellgren-Lawrence
BMI: Body mass index
ROC: Receiver operating characteristic
AUC: Area under the ROC curve
AIC: Akaike’s information criterion
